# Advances in the role of NK cells in MDS immune dysfunction and antitumor research

**DOI:** 10.3389/fimmu.2025.1511616

**Published:** 2025-03-04

**Authors:** Yinglong Wang, Zuxi Feng, Lijuan Li, Liansheng Zhang

**Affiliations:** ^1^ Lanzhou University Second Hospital, Lanzhou, China; ^2^ Department of Hematology, Lanzhou University Second Hospital, Lanzhou, Gansu, China

**Keywords:** NK cell, myelodysplastic syndromes, tumor microenvironment, immunotherapy, review

## Abstract

MDS is a heterogeneous group of myeloid neoplasms originating from hematopoietic stem cells, with a high risk of transformation into acute myeloid leukemia (AML). Natural Killer (NK) cells, crucial for their role in immune surveillance and efficient tumor cell lysis, experience functional impairments due to the complex microenvironment and cytokine dynamics in MDS. This article focuses on the mechanisms of NK cell dysfunction in MDS and the latest strategies to enhance NK cell activity to restore their anti-MDS efficacy, highlighting their key role and potential in MDS therapy.

## 1 Introduction

Myelodysplastic syndrome (MDS), a heterogeneous clonal disorder arising from hematopoietic stem cells, is characterized by the aberrant differentiation and maturation of myeloid cells, resulting in ineffective hematopoiesis, refractory cytopenias, and a propensity for progression to acute myeloid leukemia (AML). The incidence of AML increases with age, and in China, it is estimated that over 300,000 new cases are diagnosed annually, representing a substantial disease burden. As the understanding of immune mechanisms deepens, immunotherapy has gained increasing importance in the treatment of hematological malignancies, including antibody-based therapies, immune checkpoint inhibitors, tumor vaccines, and cell-based therapies such as chimeric antigen receptor T-cell (CAR-T) and natural killer (NK) cell therapies.

Natural killer (NK) cells, as crucial immune effectors, play a pivotal role in the efficient elimination of tumor cells and in maintaining immunosurveillance through the regulation of activating and inhibitory receptors. In the complex microenvironment of MDS, however, the functionality and immunosurveillance capacity of NK cells are markedly impaired due to the dynamic alterations in cytokine levels. This impairment not only diminishes the body’s ability to effectively control MDS but also potentially accelerates disease progression. In recent years, the use of NK cells in the treatment of MDS has garnered increasing attention. This paper aims to review the current research on the immunosuppressive mechanisms of NK cells in MDS patients and the associated therapeutic strategies, providing a theoretical foundation and support for future immunotherapy research.

## 2 Overview of NK cells

Natural killer (NK) cells are cytotoxic innate lymphoid cells. Based on the expression of CD56 and CD16 markers, human peripheral NK cells can be categorized into two distinct subpopulations: CD56dimCD16+ and CD56brightCD16−. The CD56dim subpopulation, which constitutes the majority of circulating NK cells, is well-known for its potent cytotoxic activity and the secretion of a wide array of immunomodulatory cytokines. Upon activation, these cells upregulate CD16 expression and mediate antibody-dependent cellular cytotoxicity (ADCC). In contrast, the CD56bright subpopulation is less mature and exhibits lower cytotoxic potential but is highly proficient in cytokine production, playing a crucial role in immunoregulation (1).

Natural killer cell receptors (NKRs), which include both activating and inhibitory receptors on the surface of NK cells, differ from T-cell receptors in that they are germline-encoded and do not undergo the complex V(D)J recombination process (1). Activating receptors include natural cytotoxicity receptors (NCRs), NKG2D, CD244, DNAM-1 (CD226), activating killer cell immunoglobulin-like receptors (KIRs), and CD94/NKG2C. Inhibitory receptors include inhibitory killer cell immunoglobulin-like receptors (iKIRs), CD94/NKG2A, and leukocyte immunoglobulin-like receptors (LILRs) with ITIM domains, which recognize non-MHC ligands (2).

**Figure 1:**
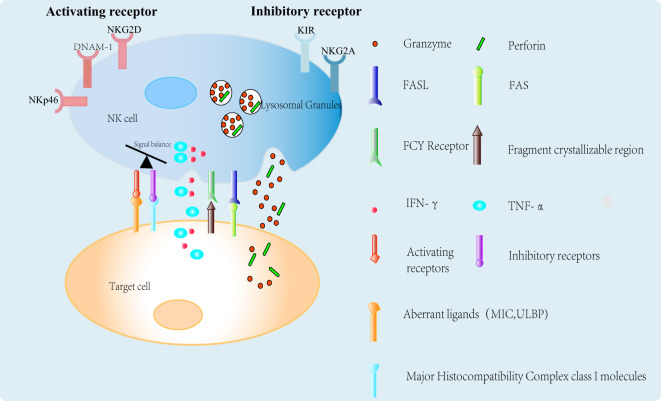
NK cell-mediated tumor killing mechanisms.

The interaction between NK cells and their ligands triggers signaling through NK cell receptors (NKRs), determining whether the NK cells are activated or inhibited. For example, healthy cells typically express low levels of ligands for activating NK cell receptors and high levels of major histocompatibility complex class I (MHC I) molecules, which bind to inhibitory killer cell immunoglobulin-like receptors (iKIRs) on NK cells, thereby protecting them from NK cell attack. In contrast, tumor cells often upregulate the expression of ligands for activating receptors, leading to the release of cytotoxic granules and cytokines by NK cells, resulting in the destruction of tumor cells (3). The specific mechanisms by which NK cells kill tumor cells are illustrated in **Figure 1**. NK cells initiate activation signals via activating receptors that recognize aberrant ligands, such as MICA/B and ULBP, on the surface of tumor cells. Simultaneously, inhibitory receptors like iKIRs interact with MHC class I molecules, which are frequently downregulated in tumor cells, thereby reducing inhibitory signals and facilitating NK cell activation.Upon activation, NK cells induce apoptosis in target cells by releasing perforin and granzymes through the immune synapse. Additionally, they secrete cytokines such as interferon-gamma (IFN-γ) and tumor necrosis factor-alpha (TNF-α), which can directly affect tumor cells or activate other immune cells by engaging death receptors on target cells via Fas ligand (FasL) or TNF-related apoptosis-inducing ligand (TRAIL).In the presence of antibodies, NK cells recognize the Fc region of the antibody via the Fcγ receptor, leading to antibody-dependent cellular cytotoxicity (ADCC) (4).

## 3 Immune dysfunction of NK cells in MDS

Natural killer (NK) cells, capable of directly eliminating cancer cells without prior sensitization, represent a highly promising immunotherapeutic approach. In patients with myelodysplastic syndromes (MDS), NK cells frequently exhibit dysfunction, characterized by decreased cell numbers, altered antigen expression, and diminished functional capacity. Montes et al. (5) demonstrated that MDS-derived NK cells, when stimulated with IL-2 and K562, secreted TNF-α and IFN-γ. Conversely, Carlsten et al. (6) observed that MDS-derived NK cells exhibited reduced degranulation and a significant decrease in cytotoxic activity following exposure to K562 leukemia target cells. These findings underscore the critical role of NK cell anti-tumor function in the disease response of MDS patients. However, the efficacy of NK cells is constrained by various factors within the tumor microenvironment, thereby influencing disease progression. Therefore, it is essential to thoroughly investigate the mechanisms underlying NK cell dysfunction in MDS, as this knowledge is crucial for enhancing the therapeutic potential of NK cells in treating MDS.

### 3.1 Tumor immune microenvironment in MDS

**Figure 2:**
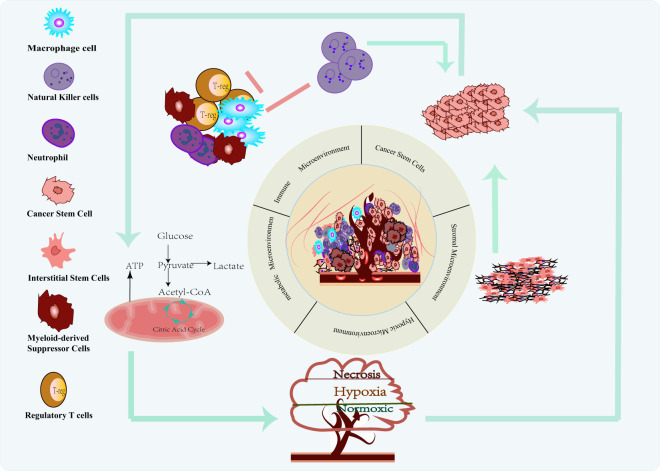
Tumor microenvironment (TME) suppressing NK cells in MDS patients.

Tumor formation results from the interaction of cancer cells with infiltrating immune cells, mesenchymal stromal cells, blood vessels, extracellular matrix (ECM), secreted products (e.g., cytokines, chemokines, metabolites), and specific environmental conditions (e.g., hypoxia). Together, all these components form a biological network that we call the tumor microenvironment (TME). In the context of myelodysplastic syndromes (MDS), the specialized tumor microenvironment (TME) exhibits a significant inhibitory effect on NK cells. Various components of the TME, including other immune cells and cytokines, work together to influence NK cell function (7). Therefore, exploring the mechanism by which the TME inhibits NK cell function is crucial for breaking through tumor immune escape and optimizing therapeutic strategies as shown in **Figure 2**.

### 3.2 Inhibition of NK cells by the immune microenvironment

NK cells are very active against tumors but are sensitive to the inhibitory components of the TME, and previous studies have shown that Tregs have an inhibitory effect on NK cells (8). Treg cells interfere with the function of NK cells through several pathways, including down-regulation of NKG2D expression in an *in vitro* co-culture model through TGF-β induction (9). Ghiringhelli et al. have found a mechanism for Treg-NK interactions, where Tregs converted growth factor-β through membrane binding to directly inhibit NK cell responses, and the absence of Tregs restored the ability of NK cells to mediate human cancer cell lysis. Notably, in the tumor microenvironment, excess ATP can be degraded by CD39 to cAMP, which is subsequently converted to adenosine by CD73 expressed on the surface of regulatory T cells (Tregs), which binds to the A2A adenosine receptor (A2AR) on NK cells, thereby negatively regulating the maturation and anti-tumor response of NK cells. Furthermore, Tregs were found to inhibit NK cells based on cytokine levels such as depletion of IL-2 (10).

In the tumor microenvironment, the coexistence of NK cells and neutrophils implies an interaction between the two. This interaction is enabled by soluble mediators, direct cell contact, and extracellular vesicles (11). In a mouse model of colorectal cancer (CRC), neutrophils reduce NK cell infiltration by down-regulating the expression of CCR1 and simultaneously inhibit the responsiveness of the NK cell activation receptors NKp46 and NKG2D (12). Furthermore, the interaction between neutrophils and NK cells is a key component in promoting the invasion-metastasis cascade response. In a mouse model injected with 4T1 breast cancer cells, neutrophils inhibited NK cell activity, thereby attenuating NK cell clearance of intravascular cancer cells (13). It is suggested that neutrophils (NETs) have the ability to block the killing effect of NK cells on tumor cells and provide evidence that NETs interfere with the contact of NK cells with cancer cells (14).

Macrophages are the most abundant subpopulation of immune cells in a wide range of tumor types and are directly or indirectly involved in several key features of malignancy, including angiogenesis, metastasis, immunosuppression, and treatment resistance (15). Macrophages and NK cells crosstalk through different mechanisms in which soluble mediators play a central role. In the tumor microenvironment, macrophages inhibit NK cell activation receptors (NKG2D and NKP30) by secreting transforming growth factor-β, modulate chemokine receptor (CX3CR1) expression, and promote the expression of inhibitory receptors (ILT-2), which in turn reduces the release of cytokines (interferon-gamma and tumor necrosis factor-α) from NK cells (16).

### 3.3 Inhibition of NK cells by the stromal microenvironment

Fibroblasts (CAFs) are one of the most important stromal components of the tumor microenvironment and can be derived from multiple cell types at different stages of differentiation in different parts of the body. CAFs are able to inhibit NK-cell-mediated cancer cell death in a variety of ways, including the expression of soluble mediators, regulation of NK-cell-activated receptors, and synergistic interactions with other immune cells (17). Fibroblasts effectively inhibited NK cell-mediated cell death by inhibiting the expression of poliovirus receptor (PVR/CD155) or by promoting the recruitment of M2-type macrophages, reflecting their role in regulating NK cell function (18). The importance of transforming growth factor-β (TGF-β) as a core cytokine mediating the interaction between cancer-associated fibroblasts (CAFs) and natural killer (NK) cells is becoming increasingly significant. Previous studies (19) have shown that TGF-β inhibits NK cell function mainly by interfering with the secretion of cytokines (e.g., IFN-γ) and down-regulating the expression of NK cell-activating receptors (e.g., NKG2D, NKp30, and NKp44).

### 3.4 Suppression of NK cells by bone marrow mesenchymal stem cells

Bone marrow mesenchymal stem cells (MSCs), as pluripotent stem cells, have the ability to differentiate into a wide range of mesenchymal lineages, which makes them a key factor in regulating tumor fate (20). Existing studies have confirmed the inhibitory effect of MSCs on NK cells. MSCs inhibit NK cell function by producing and releasing soluble factors such as transforming growth factor β (TGF-β), prostaglandin E2 (PGE2), indoleamine 2,3-dioxygenase (IDO), human leukocyte antigen G (HLA-G), and exosomes (21); the direct interaction with the NK cell contact, which can inhibit NK cell function by down-regulating the expression of activated receptors on the surface of NK cells or reducing perforin release, as well as by interfering with intracellular signaling pathways of NK cells and interactions with other immune cells (Treg cells) (22).

### 3.5 Suppression of NK cells by cancer stem cells (CSCs)

Dormant stem cells hidden within tumors, called cancer stem cells (CSCs), are characterized by their self-renewal capacity and multidirectional differentiation. NK cells can recognize and lyse CSCs, but the lysogenic effect of natural killer cells (NK cells) on cancer stem cells (CSCs) is controversial. Al-Hajj et al. (23) showed that, in NOD/SCID mice CSC subpopulations are more likely to trigger tumorigenesis than non-CSCs, and that CSCs may be more phenotypically equipped to resist NK cell-mediated cell death to evade NK cell surveillance. The mechanism by which CSCs evade NK cell surveillance involves their up-regulation of major histocompatibility complex class I (MHC-I) molecules, thereby gaining resistance to NK cells (24). Previous studies (25) found that human leukocyte antigen G (HLA-G) and HLA-E send inhibitory signals to NK cells upon binding of ligands, which is partially manifested by immune evasion in CSCs. In breast cancer (BC), CSCs resist NK cell-mediated cell death by disrupting the balance between NK-activating ligands and inhibitory receptors (26).

### 3.6 Suppression of NK cells by the metabolic microenvironment

Cancer cells generate a heterogeneous tumor microenvironment (TME) through multiple metabolic pathways as a means of evading immune surveillance by natural killer cells (NK cells) (27). Widespread hypoxia in the TME has been suggested to be a prevalent factor in tumor immune escape. Under hypoxic conditions, NK cells undergo functional reprogramming, which profoundly affects the infiltrative properties and immune-mediated responses of NK cells within the tumor tissue (28). Mitochondrial dysfunction, down-regulation of NK cell-activated receptors, reduction of perforin, and degradation of NK cell-derived granzyme B are the key mechanisms underlying the impaired effector function of NK cells in hypoxic environments (29).

Glucose is a key energy source for cytotoxic natural killer cells (NK cells), which rely on glucose-driven glycolysis and oxidative phosphorylation (OXPHOS) to meet their energy requirements (30). Increasing evidence suggests that the high glycolytic capacity and glycolytic reserve of NK cells are essential to support their anti-tumor activity. Dysregulated cellular energy metabolism is another important feature of cancer cells (26). Under aerobic conditions, cancer cells utilize glucose for glycolysis, a process known as aerobic glycolysis or the Warburg effect. Under metabolic stress, cancer cells inevitably compete with NK cells for limited glucose resources, and tumor-induced glucose restriction may reduce the glycolytic efficiency of NK cells, which in turn impairs their anti-tumor function (31).

Lactic acid produced by glucose consumption in hypoxic tumor regions through anaerobic glycolysis could serve as an energy source for the tricarboxylic acid cycle (TCA cycle) in neighboring cancer cells (32). Although cancer cells can respond to extracellular acidification and redox reactions by regulating the expression of monocarboxylic acid transporter proteins (MCTs) to adapt to survival and proliferation, exposure to high lactate environments impairs the effector function of natural killer cells (NK cells). Preliminary evidence suggests that lactate can impair NK cell function and survival, leading to immune escape and tumor progression. Tumor-derived lactate can inhibit the cytotoxic function of NK cells through direct or indirect pathways involving decreased expression of NK cell perforin, granzyme, NKp46, and increased abundance of myeloid-derived suppressor cells (MDSCs) (33).

## 4 Suppression of NK cell function by MDS-associated gene mutations

In the context of myelodysplastic syndromes (MDS), the function of natural killer (NK) cells is often suppressed even though their number remains stable, suggesting a potential effect of MDS-associated gene mutations on NK cell activity. In a study (34), 33 MDS/chronic myelomonocytic leukemia (CMML) patients carrying TET2/IDH mutations and 23 healthy controls were analyzed by flow cytometry for NK cell surface markers, including maturation markers (CD69, CD57, KLRG1), activating receptors (NKp30, NKp46, DNAM-1, NKG2D) and inhibitory receptors (CD96, KIR2D, CD85j), the results showed that the expression of some activating (e.g., DNAM-1, NKp46) and inhibitory receptors (CD96) in NK cells from MDS/CMML patients was subtly altered, and that the expression of KIR was significantly decreased to 31.2% in contrast to 54.8% in healthy controls (p<0.01). In-depth investigation revealed that impaired NK cell function in MDS patients may be associated with epigenetic modifications such as aberrant JAK/STAT signaling and DNA methylation, revealing the molecular mechanism of NK cell dysfunction in MDS (35).

## 5 Advances in immunotherapy for MDS using NK cells

In the treatment of myelodysplastic syndromes (MDS), allogeneic hematopoietic stem cell transplantation (HSCT) is currently considered the only curative option. However, relapse of leukemia after transplantation is a major factor contributing to treatment failure. Effective treatment options are extremely limited for those patients who relapse or are resistant to existing therapies. Given the clinical efficacy demonstrated by natural killer (NK) cell immunotherapy in solid tumors and hematological tumors, it is worth exploring its application in the treatment of MDS.

### 5.1 Adoptive immunotherapy

#### 5.1.1 CAR-NK

Compared to CAR-T cell therapy, CAR-NK cell therapy allows unlimited use of allogeneic NK cell sources without concern for the development of Graft-Versus-Host Disease (GVHD), as well as the potential to produce ‘off-the-shelf’ products using NK cell lines or induced pluripotent stem cell (iPSC)-NK cells. ‘off-the-shelf’ products, in addition to relatively short preparation times, recognition and killing of tumor cells via receptors inherent to NK cells, thus reducing the possibility of disease escape due to downregulation of CAR antigens, a phenomenon observed in CAR-T cell therapies (36).

Similar to CAR-T cells, CAR-NK cells are genetically modified to express chimeric antigen receptors (CARs) that recognize specific antigens that are specifically expressed or overexpressed by target cells. In most preclinical studies, lentiviral or retroviral transduction was used to achieve stable and sustained CAR expression in NK cells. In addition, non-viral vector delivery methods such as transposon systems and mRNA electroporation have also been used (37). CAR-NK cells have been targeted against a wide range of tumor antigens in preclinical studies for hematological malignancies and solid tumors (38). In one preclinical study, CD123 CAR-NK cells (5-day survival: 100%) showed lower acute toxicity than CD123 CAR-T cells (5-day survival: 0%) in a mouse model implanted with artificial blood cells, whereas, in a mouse model of acute myelogenous leukemia (AML), the two were comparable in terms of their antileukemic efficacy; another CAR for AML NK cell therapy study showed that CD33/FLT3 CAR-NK cells exhibited encouraging anti-leukemia effects in an animal model (>90% of leukemia cells were killed) and the addition of endothelial-inhibitory CARs protected approximately 42% of human healthy hematopoietic stem cells (HSCs) and hematopoietic progenitor cells (HPCs) from cytotoxicity in *in vitro* experiments damage (39).

In summary, although CAR-NK cell therapy is still in the early stages of research and development, and its long-term effects, optimal application populations, and strategies for combining with other therapies are still being explored, CAR-NK cell therapy still offers a potential new direction for MDS treatment.

#### 5.1.2 Allogeneic NK cells

One of the most striking examples of the anti-tumor function of NK cells stems from the ‘self-deficiency’ recognition mechanism. Hematopoietic stem cell transplantation (HSCT) is an effective and potentially curative treatment option for patients with myeloid hematological tumors. Allogeneic HSCT relies on HLA matching between donor and recipient to avoid graft-versus-host disease (GVHD). In the absence of an HLA-compatible donor, HLA hemizygous HSCT is performed, where the recipient and donor share only one HLA haplotype (usually one of the parents). A series of seminal studies have shown that in hemi-compatible HSCT, those recipients whose HLA molecules are mismatched to the donor KIR have a lower rate of post-transplant relapse, suggesting a strong NK-mediated graft-versus-leukemia (GvL) effect, whereas KIR mismatch did not lead to GVHD (40).

The potent GvL effect of KIR-mismatched NK cells in hemizygous HSCT has inspired hematologists to explore highly purified hemizygous NK cell infusions to enhance GvL. Clinical trials have reported complete remissions in elderly acute myeloid leukemia (AML) patients and 100% event-free survival in pediatric AML cohorts at 18-month follow-up (41).

#### 5.1.3 Autologous NK cells

Autologous NK cells have also been explored for cancer immunotherapy, but the field is not as well developed as autologous T cell transfer. Although NK cells can be isolated from the peripheral blood of patients and expanded *in vitro*, the expansion of NK cells is more challenging than that of T cells. In clinical trials, although autologous NK cells can successfully colonize and persist in the peripheral blood after transplantation, no significant clinical response has been observed (42). The functional status and expansion capacity of autologous NK cells tend to be poor. This may be due to the treatment the patient received prior to NK cell isolation, which may also explain their poor clinical outcome. To overcome this problem, researchers are exploring several strategies, including the application of different combinations of activating cytokines (IL-2, IL-12, IL-15, IL-18) and the use of feeder cells to deliver key growth factors during *in vitro* expansion (43). In this regard, a phase I trial in multiple myeloma (MM) using autologous NK cells activated by feeder cells expressing membrane-bound IL-15 and 4-1BBL showed modest clinical activity (44), suggesting that optimization of feeder cells may be helpful in improving the activation status of NK cells prior to overt metastasis. In addition, it has been shown that autologous NK cells are more effective when tumor cells lack at least one HLA ligand recognized by KIR expressed by metastatic NK cells (i.e., the ‘ligand-deficient’ hypothesis) (45).

### 5.2 Cytokine therapy

Cytokines, as key regulators of natural killer (NK) cells, are an attractive option for tumor immunotherapy. Cytokine secretion is critical for NK cell pre-activation. A variety of cytokines, including IL-2, IL-12, IL-15, IL-18, and IL-21, can be used to enhance NK cell activation (46). In autologous NK cell transfer, IL-2 is commonly used for NK cell activation and expansion. Patients receiving NK cell infusions are usually given IL-2 to promote expansion *in vivo*. However, the antitumor effect of IL-2, IL-12, or IL-18 alone is more limited. In contrast, the combination of IL-12, IL-15, and IL-18 may induce significant biological changes and generate memory NK cell populations, including enhanced responses to IL-2, greater IFN-γ secretion, and stronger cytotoxicity (47). Furthermore, the combination of IL-21 with tumor-targeting monoclonal antibodies enhances NK cell activity and improves their anti-tumor effects (48). However, among these cytokines, IL-15 is the most promising one for activating NK cells. In clinical trials in patients with metastatic malignancies, IL-15 infusion showed proliferation and a significant increase in NK cells (49).

### 5.3 Immune checkpoint inhibitors

Immunotherapy, with its growing array of therapeutic strategies, has become a powerful tool in the fight against cancer. Suppression of immune checkpoints forms the core of immunotherapy. While previous studies have focused on T cells, natural killer (NK) cells have emerged as a new target for immune intervention. Combining immune checkpoint inhibitors with enhanced anti-tumor activity of NK cells opens up new avenues in the treatment of myelodysplastic syndromes.

#### 5.3.1 PD-1

Programmed cell death-1 (PD-1) is expressed in a wide range of immune cells, including T cells (CD4 & CD8), B cells, myeloid cells, natural killer (NK) cells, NKT cells, and other intrinsic lymphocytes (IL2) (50).

The expression of PD-1 on NK cells shows diversity in different cancers. Typically, CD56bright NK cells lack PD-1 expression. However, CD56dim NK cells exhibit PD-1 expression restricted to the NKG2A- KIRCD57 phenotype, which is mature NK cells (51). NKG2A++- KIRCD57 phenotype NK cells are thought to have significantly downregulated activation receptors such as NKp30 and NKp46. There is a correlation between PD-1 expression and impaired anti-tumor activity of NK cells, and disruption of PD-1-PD-L1 interaction by antibodies can partially restore activity (50).

The upregulation of PD-1 expression in NK cells in a variety of cancers suggests that NK cells may be in a dysfunctional state, which may be due to the overstimulation of MHC-I-deficient tumor cells. Comparison of PD-1+ NK cells with PD-1- NK cells showed functional depletion of PD-1+ NK cells, which manifested itself as impaired cytotoxicity, cytokine production, and proliferative capacity (51, 52). Blockade with anti-PD-1 monoclonal antibodies (mAbs) restores the functional capacity of NK cells. Mouse tumor-resident NK cells express PD-1, whereas anti-PD-1 blockade stimulates an anti-tumor immune response in NK cells (53).

In patients with PTLD, anti-PD-1 pathway disruption enhanced IFN-γ release without affecting cytotoxicity, suggesting partial dependence on the PD-1 pathway (54). Therefore, more studies are needed to clarify the role of PD-1 blockade in the context of NK cells. Blockade of PD-L1 has also been shown to improve NK cell-based antitumor responses (55). ADCC (antibody-dependent cell-mediated cytotoxicity) against a wide range of cancer cells obtained using the anti-PD-L1 antibody avelumab was enhanced in NK cells and tumors undergoing epigenetic preconditioning (56). PD-L1-independent killing of CRC cells by densely activated predominantly human NK cells under three-dimensional culture conditions has also been demonstrated (57).

#### 5.3.2 KIRs

KIRs, each recognizing a specific human leukocyte antigen class I subtype (HLA-A, -B, or -C) as a ligand (58). Inhibitory KIR2DL1, KIR2DL2, and KIR2DL3 recognize HLA-C as a ligand, whereas the other KIRs, including inhibitory KIR3DL1 and KIR3DL2, use HLA-B and HLA-A as ligands, respectively. In addition to NK cells, T cell subsets and iNKT cells (invariant natural killer T cells) also express KIRs (59).

IPH2101 and lirilumab (IPH2102/BMS-986015) are IgG4 monoclonal antibodies (mAbs) targeting the KIR2DL1/2/3 NK inhibitory receptor, which are currently being investigated as single agents or in combination with other drugs including molecularly-targeted agents (lenalidomide), monoclonal antibodies (elotuzumab & rituximab) and immune checkpoint inhibitors (ibritumomab & navumumab)) have been clinically evaluated and developed (60). ipH2101 has been clinically evaluated in a variety of hematological systems (acute myeloid leukemia, chronic lymphocytic leukemia, non-Hodgkin’s lymphoma) (61). A recent study reported the efficacy and tolerability of lirilumab as monotherapy or in combination with azacitidine in patients with myelodysplastic syndromes (MDS) (62).

## 6 Summary

Cellular immunotherapy stands at the forefront of cancer research, and the success of chimeric antigen receptor T (CAR-T) cell therapy has spurred the development of innovative cellular therapeutic approaches for both hematological malignancies and solid tumors. Natural killer (NK) cells represent an alternative cellular source that shares similar effector functions with T cells, yet offers lower toxicity and the potential for off-the-shelf use. Notably, in contrast to T cells, the use of NK cells in allogeneic transplantation does not typically lead to cytokine release syndrome (CRS) or graft-versus-host disease (GVHD). In hematological malignancies, the anti-tumor activity of NK cells can be compromised by both targeted cancer therapies and the tumor microenvironment. Consequently, enhancing NK cell function is crucial for improving the outcomes of hematological myeloid tumor treatments.

However, the inhibitory effects of the tumor microenvironment and MDS-associated genetic mutations on NK cell numbers and function impair the ability of NK cells to perform immunosurveillance and eliminate tumor cells. To tackle this challenge, we have undertaken research to elucidate the specific mechanisms underlying NK cell suppression within the tumor microenvironment and to investigate strategies for enhancing the cytotoxic activity of NK cells against tumor cells. These investigations lay a crucial scientific foundation for further exploration of the therapeutic potential of NK cells in the treatment of myelodysplastic syndromes.
